# Piezoelectric Potential in Single-Crystalline ZnO Nanohelices Based on Finite Element Analysis

**DOI:** 10.3390/nano7120430

**Published:** 2017-12-07

**Authors:** Huimin Hao, Kory Jenkins, Xiaowen Huang, Yiqian Xu, Jiahai Huang, Rusen Yang

**Affiliations:** 1 Key Lab of Advanced Transducers and Intelligent Control System, Ministry of Education and Shanxi Province, Taiyuan University of Technology, Taiyuan 030024, China; xu276816496@sina.com (Y.X.); huangjiahai@tyut.edu.cn (J.H.); 2 Department of Mechanical Engineering, University of Minnesota, Minneapolis, MN 55455, USA; jenk0131@umn.edu; 3 Department of Applied Physics, The Hong Kong Polytechnic University, Hong Kong, China; huangxiaowen2013@gmail.com; 4 School of Advanced Materials and Nanotechnology, Xidian University, Xi’an 710071, China

**Keywords:** piezotronic, numerical simulation, nanohelix, FEM

## Abstract

Electric potential produced in deformed piezoelectric nanostructures is of significance for both fundamental study and practical applications. To reveal the piezoelectric property of ZnO nanohelices, the piezoelectric potential in single-crystal nanohelices was simulated by finite element method calculations. For a nanohelix with a length of 1200 nm, a mean coil radius of 150 nm, five active coils, and a hexagonal coiled wire with a side length 100 nm, a compressing force of 100 nN results in a potential of 1.85 V. This potential is significantly higher than the potential produced in a straight nanowire with the same length and applied force. Maintaining the length and increasing the number of coils or mean coil radius leads to higher piezoelectric potential in the nanohelix. Appling a force along the axial direction produces higher piezoelectric potential than in other directions. Adding lateral forces to an existing axial force can change the piezoelectric potential distribution in the nanohelix, while the maximum piezoelectric potential remains largely unchanged in some cases. This research demonstrates the promising potential of ZnO nanohelices for applications in sensors, micro-electromechanical systems (MEMS) devices, nanorobotics, and energy sciences.

## 1. Introduction

Helical structures have been widely used in industry due to their low stiffness and superior capability to resist large axial strain, while helical structures are also the fundamental configuration for DNA and many other biomolecules. Three-dimensional helical nanostructures of zinc oxide (ZnO) have also been investigated, such as nanohelices [1,2,3], nanorings formed by self-coiling nanobelts [4,5], and nanosprings [6,7,8]. ZnO helical nanostructures showed superelastic behavior, and their spring constant increased continuously up to 300–800% when they were stretched [2]. As a piezoelectric material, ZnO nanostructures generate piezoelectric potential when exposed to physical stimulation, such as stretching, compression, and bending. Taking advantage of this phenomenon, ZnO has been used to fabricate nanogenerators for energy harvesting [9,10,11,12]. ZnO is also a semiconductor material. Piezoelectric potential can alter electronic transport in ZnO nanostructures, which has resulted in novel devices, e.g., piezoelectric field effect transistors [13], strain sensors [14,15], programmable electromechanical memories [16], and logic circuits [17].

Considering the importance of piezoelectric potential in ZnO nanostructures for their applications in electronics, sensors, actuators, and nanogenerators, the distribution and effects of piezoelectric potential in ZnO nanowires have been studied. Lippman theory [18,19,20] has been applied to predict the distribution of equilibrium potential in a deformed ZnO nanowire. Wang calculated the piezoelectric potential in a bent ZnO nanowire, and a numerical calculation of the piezoelectric potential distribution in a ZnO nanowire without doping was carried out. The potential difference was around 0.4 V in a ZnO nanowire grown along the *c*-axis with a length of 1200 nm and a hexagonal side length of 100 nm under a compressing force of 85 nN [9]. The calculation results were verified by later measurements of the asymmetric voltage distribution on the tensile and compressive side surfaces of a ZnO nanowire [9]. Experimental measurements and numerical modeling works revealed that the piezoelectric potential can cause a re-distribution of free charge carriers in ZnO nanowires and modify the electronic transport of ZnO nanowire-based devices. However, for the piezoelectric potential of ZnO, current studies are mainly limited to nanowires.

Owing to the enriched physical and chemical properties, the unique spiral geometry of helical nanostructures was studied to discover novel properties for new nanodevice design and fabrication. Chen studied the mechanics of carbon nanocoils. They found that the spring constant K stays constant with increasing elongation when the spring had lower helical angles, and that the carbon nanocoil returned completely to its relaxed geometry after loading without apparent plastic deformation [21]. For potential device applications, the mechanical properties [22,23], electrical properties [24,25,26], optical properties [27,28], and magnetic properties [29] of helical nanostructures of several materials were studied. Nevertheless, the study of piezoelectric potential in helical nanostructures is still lacking.

Ultrasmall, deformation-free, single-crystal nanohelices of piezoelectric ZnO have been reported [1]. It has been shown that the electrostatic interaction between polar surfaces plays an important role in forming the deformation-free nanohelices. In this work, we used a three-dimensional (3D) finite element method (FEM) to examine the piezoelectric potential in ZnO nanohelices. We discussed the effects of the number of coils, mean radius of the nanohelix, and applied forces on the piezoelectric potential. We focused on the equilibrium piezoelectric potential and showed that nanohelices could produce significantly higher potential than a nanowire with the same height under the same force. These results indicate that ZnO nanohelices can be excellent candidates for fabricating piezoelectric nanodevices, such as nanogenerators, actuators, and nanosensors.

## 2. Model and Method

### 2.1. Model Configuration

**Figure 1:**
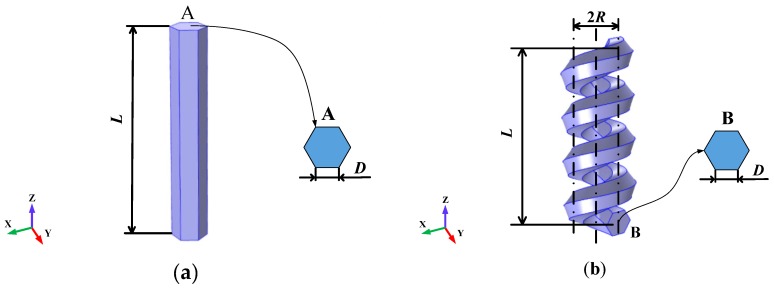
Schematic illustration of (**a**) ZnO nanowire and (**b**) ZnO nanohelix. Both nanostructures have a hexagonal cross-section with the same side length *D* and height *L*. The nanohelix has a number of coils *T* and a mean radius of coil *R*.

Figure 1 shows the structure of a ZnO nanowire and a ZnO nanohelix. The ZnO nanowire in Figure 1a is modeled as a cylinder with a hexagonal cross-section in the a-b crystallographic plane and its *c*-axis along the *z*-axis; Figure 1b shows a deformation-free, single-crystal nanohelix of piezoelectric ZnO with a hexagonal cross-section in the a-c crystallographic plane and its *c*-axis along the *z*-axis. Nanowires are normally grown along their *c*-axis, which is along the *z*-axis in Figure 1a. The ZnO nanohelix in Figure 1b is formed by a wire with a hexagonal cross-section. The center line of the ZnO nanohelix coincides with the *z*-axis. A sequential rotation in the growth direction results in a non-twisted single-crystal structure for the entire nanohelix [1]. The details of the growth and structure characterization of the nanohelix are reported in Reference [1]. The hexagonal side length D and total length along the major axial direction L are assumed to be the same for the nanowire and the nanohelix. The mean radius of the coil and the number of coils of the nanohelix are represented by *R* and *T*, respectively. Studies of right-handed nanohelices and left-handed nanohelices produced similar results, and the results of right-handed nanohelices are presented here. 

We assumed that there is no body force $f_{e}^{b}$ and no free charge carriers $\rho_{e}^{b}$ in order to simplify the computation and focus on the piezoelectric potential created in the nanowires and nanohelices. The effect of free charge carriers in nanowires has been investigated and reported [19,30]. A more comprehensive evaluation of piezoelectric nanohelices with free charge carriers considered is the subject of a future study. The modeling parameters are given in Table 1 [31].

### 2.2. FEM Modeling of Nanowires and Nanohelices

Since there is no body force in the nanostructures, the divergence of the stress tensor σ should be zero in the static piezoelectric problem [19,20]:

(1) $$\nabla \cdot \sigma ={\vec{f}}_{e}^{b}=0$$

where σ is the stress tensor.

The constitutive relation between stress σ and electric displacement $\vec{D}$ is governed by the fundamental piezoelectric equations:

(2) $$\{\begin{array}{c} \sigma_{p}=c_{pq}\varepsilon_{q}-e_{kp}E_{k}\\ D_{i}=e_{iq}\varepsilon_{q}+\kappa_{ik}E_{k} \end{array}$$

where $c_{pq}$ is the linear elastic constant, ε is the strain, $e_{kp}$ is the linear piezoelectric coefficient, $\kappa_{ik}$ is the dielectric constant, and E is the electric field.

By assuming no free charge $\rho_{e}^{b}$ in the nanowire or the nanohelix, the Gauss equation must be satisfied:

(3) $$\nabla \cdot \vec{D}=\rho_{e}^{b}=0$$

and the compatibility equation should be satisfied:

(4) ∇×∇×ε=0

We calculated the piezoelectric potential in nanowires and nanohelices by solving the above nonlinear partial differential Equations (1)–(4) with the program COMSOL Multiphysics^®^. The bottom face of the nanowire and the nanohelix was fixed and electrically grounded in our model. A force was applied only to the top end, and the piezoelectric potential was numerically calculated. A nanowire and a nanohelix with the same side length and height were simulated to compare their piezoelectric potentials when exposed to the same force. A series of nanohelices with different numbers of coils and mean radii of the coil were calculated to reveal the change of piezoelectric potential in a helical structure. The effect of force direction was also investigated.

## 3. Simulation Results and Discussion

### 3.1. Pizeoelectric Potential and Displacement of the ZnO Nanohelix and Nanowire

**Figure 2:**
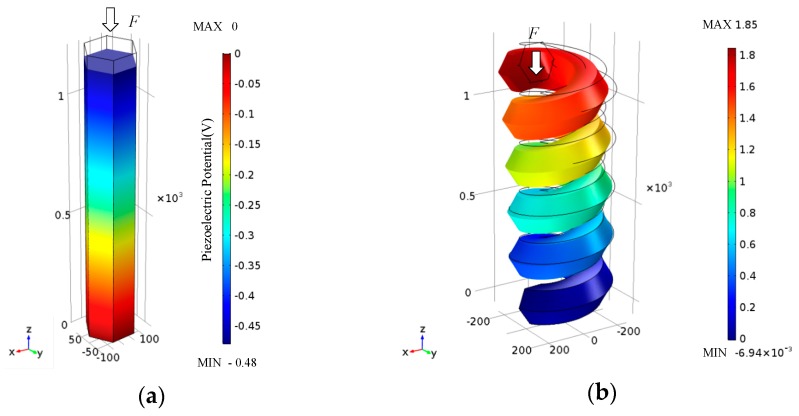
The piezoelectric potential distribution in (**a**) ZnO nanowire and (**b**) nanohelix under a compressing force of 100 nN along the *z*-axis.

The piezoelectric potential has been extensively calculated for ZnO nanowires, and the fabrication of nanowire-based nanogenerators has seen great success. It is of great interest to know the piezoelectric potential in other nanostructures. In our initial study, we considered a nanowire and a nanohelix with the same side length D = 100 nm and height L = 1200 nm. The nanohelix had five coils and a mean radius of coil of *R* = 150 nm. A compressing force of *F* = 100 nN was applied to the nanowire at the top surface along the *z*-axis, and the same force of *F* was also applied on the upper cross-section of the nanohelix parallel to the *z*-axis, such that the nanowire and the nanohelix were compressed. Calculated piezoelectric potentials in the nanowire and the nanohelix are shown in Figure 2. 

In Figure 2a, the red side is ground and the blue side is the negative potential side. The nanowire in Figure 2a shows a maximum piezoelectric potential of 0.48 V in the nanowire and the top surface shows a maximum displacement of 0.03 nm. A maximum piezoelectric potential around 0.4 V was obtained when we applied an 85 nN compressing force in the same way. This is consistent with a previous work, and lends confidence to our simulation results [9]. The nanohelix has a greatly reduced stiffness due to its helical structure. In Figure 2b, the blue side is ground and the red side is the positive potential side. The maximum displacement of the nanohelix in Figure 2b reached 10.2 nm under the same 100 nN compressing force. The numerical computation revealed a maximum piezoelectric potential of 1.85 V at the top of the nanohelix, which is significantly greater than the piezoelectric potential of 0.48 V found in the nanowire. Owing to the single-crystal structure, the same height ZnO nanohelix can be thought of as a longer ZnO nanowire. By the piezoelectric potential, the piezoelectric field is created through the constructive add-up of the dipole moments created by all units in the crystal. More units will create a higher piezoelectric potential. Therefore, the piezoelectric potential continuously drops from one end of the nanohelix to the other. Meanwhile, numerical calculation of the piezoelectric potential distribution in a ZnO nanohelix at a stretching force of 100 nN was calculated. Note that the stretching force generated the same continuous piezoelectric potential in the ZnO nanohelix with reversed polarity.

This work shows that the nanohelix can produce higher potential than the nanowire when they are exposed to the same force. In addition, the helical structure has a much lower resonant frequency along its central axial direction than that of the nanowire. Consequently, nanohelix-based nanogenerators may perform better than nanowire-based nanogenerators to harvest energy from the environment where low-frequency vibration is more frequently observed.

### 3.2. Effect of the Number of Coils and the Mean Radius of the Coil on the Pizeoelectric Potential of a Nanohelix

**Figure 3:**
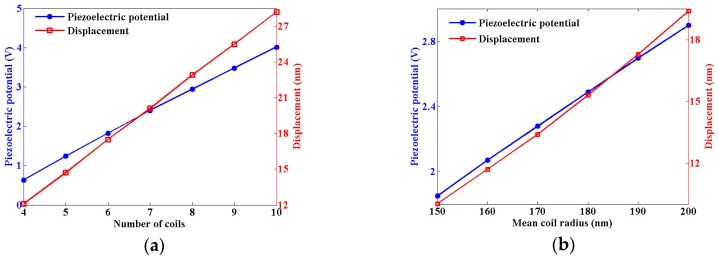
The change of the maximum piezoelectric potential and displacement with the number of coils in (**a**) and the mean coils radius in (**b**) of ZnO nanohelices with a constant length of 1900 nm.

A nanohelix is normally formed from a nanowire that grows in a specific crystallographic direction and follows a helical path [1]. Different growth conditions can result in nanohelices of different dimensions. For the research of a greater number of coils, we used a second model, similar to the first but with an increased length of 1900 nm. We thus studied the deformation and the piezoelectric potential produced from different nanohelices with the same length of 1900 nm. We kept the mean coil radius at 150 nm and changed the number of coils from four to 10. The spring pitch as well as the stiffness decreased as the number of coils increased. We applied a compressive force of 100 nN along the *z*-axis at the top face of the nanohelix. The piezoelectric potential in the nanohelices was calculated using FEM simulation. The maximum potential was found at the top part of the nanohelix in this study, and the potential increased from 0.63 V to 4.01 V as the number of coils increased. The displacement increased from 12.1 nm to 28.2 nm with the increasing number of coils, as shown in Figure 3a. We then kept the number of coils at five and changed the mean coil radius of the nanohelix. Our simulation of nanohelices with different mean coil radius revealed a similar trend. As the mean coil radius increased from 150 nm to 200 nm under the same applied force of 100 nN, the piezoelectric potential in the nanohelix increased from 1.85 V to 2.90 V, while the displacement of the nanohelix increased from 10.02 nm to 19.40 nm, as shown in Figure 3b. 

Figure 3a shows that the displacement and the piezoelectric potential increased linearly with the number of coils, as expected. Figure 3b shows that the piezoelectric potential increased linearly with the mean coil radius, while nonlinearity was found in the displacement. Due to the special helical structure, the total displacement of the nanohelix in Figure 3b consists of three components including the displacement in the *x*-direction, displacement in the *y*-direction, and displacement in the *z*-direction. Previous researchers showed that the spring constant K of a linear elastic spring was directly proportional to $1/R^{3}$ (where R is the coil radius), if the force was applied along the central axis and only the torsion generated from the extension of the spring in the low-strain regime was considered [32,33]. Consequently, when a force *F* along the *z*-axis is applied to a typical spring with *d* ≪ R, a nonlinear behavior was observed in displacements ($\Delta =F/K\propto R^{3}$), which is consistent with our result shown in Figure 3b. However, the diameter of the coil wire was comparable to the mean coil radius of the helix in this work, and the location of the force applied was not on the central axis of the helix. The difference in the structure and the force applied in this work compared to an earlier study [32,33] resulted in a small deviation of the behavior shown in Figure 3b from the behavior of a typical spring.

### 3.3. Effect of Acting Forces on the Pizeoelectric Potential and Displacement

Different forces may cause nanowires to be bent, stretched, or compressed. The piezoelectric potential in a bent nanowire was first used in nanogenerators and piezoelectric potential field effect transistors [9,10,13]. Later, the piezoelectric potential in a stretched or compressed nanowire was used for the fabrication of nanogenerators [11,34], tactile imaging [35], sensors [36], and other devices [37]. Nanohelices produced greater piezoelectric potential than nanowires as they were compressed along the axial direction. We further studied the piezoelectric potential in a nanohelix as different forces were applied.

To simplify the calculation, all of the compressing forces were uniformly applied at the upper face of the nanohelix. We calculated the piezoelectric potential of the nanohelix for separate cases where a force of 100 nN was applied along the *x*-axis, *y*-axis, or *z*-axis. We also calculated the piezoelectric potential of the nanohelix under different combinations of these forces. The maximum potential in each case is summarized in Table 2. The force along the *z*-axis clearly produced higher piezoelectric potential than the same amount force applied in other directions, and the displacement was also at its minimum when the force was only along the *z*-axis. When a lateral force was applied, the nanohelix was bent significantly. The force in the *x*-direction pushed the top end towards the central axis of the spring and the produced maximum piezoelectric potential was lower than that produced by a force in the *y*-axis. When two or more force components were involved, the maximum piezoelectric potential was normally higher with a force along the *z*-axis included.

When a lateral bending force was added to the existing compressing force along the *z*-axis, the piezoelectric potential distribution was significantly changed. However, the maximum piezoelectric potential in the nanohelix could change in any direction because the maximum and minimum potentials due to each individual force component occurred at different locations. For example, the maximum piezoelectric potential was 1.85 V when a force of 100 nN was applied along the *z*-axis. The potential remained at 1.85 V as an additional force of 100 nN along the *x*-axis was added. In comparison, the maximum piezoelectric potential decreased to 1.60 V when a force of 100 nN along the *y*-axis was added. This result indicates a coupling effect between the three applied force components on the generation of piezoelectric potential.

The displacement of the nanohelix is dependent on its mechanical property and the boundary condition. In our case, the fixed constraint is on the *x*-*z* plane. Owing to the stiffness of the nanohelix, the displacement along the *x*-axis and *y*-axis are greater than that along the *z*-axis. In particular, deformation along the *y*-axis is most prone to deform the spring. Similarly, the coupling effect between the three directions of applied force components on the total displacement cannot be ignored, even though the total displacement is the synthesis of the *x*-axis, *y*-axis, and *z*-axis components.

## 4. Conclusions

In conclusion, FEM simulation has been used to study the piezoelectric potential in a ZnO nanohelix, and it predicted much higher piezoelectric potential than that in a nanowire with the same length and applied force. Increasing the number of coils or mean coil radius of a nanohelix of a constant height results in a higher maximum piezoelectric potential when the force is kept constant. Both lateral bending force and vertical compressing force can create piezoelectric potentials. Applied forces in different directions have a coupling effect on the piezoelectric potential. A force along the *z*-axis produces a higher maximum piezoelectric potential that favors energy harvesters and other piezotronic devices. Adding a lateral force to the existing vertical force can change the distribution of the piezoelectric potential while the maximum potential may not be greatly changed. Meanwhile, the mechanical property of a ZnO nanohelix is also studied. The force component on the *y*-axis showed the biggest effect on displacement. This work not only demonstrated a new and excellent candidate for nanogenerators and other electronic devices, but it is also expected to lead to new exploration of other piezoelectric nanostructures.

## Figures and Tables

**Table 1:** List of parameters for modeling.

Parameters	Value
Density (kg/m^3^)	5680
Elastic constants	
*c*_11_ (GPa)	209.7
*c*_12_ (GPa)	121.1
*c*_13_ (GPa)	105.1
*c*_33_ (GPa)	211.3
*c*_44_ (GPa)	42.3
*c*_55_ (GPa)	43.6
Piezoelectric constants	
*e*_31_ (C/m^2^)	−0.57
*e*_33_ (C/m^2^)	1.32
*e*_15_ (C/m^2^)	−0.48
Relative dielectric constants	
$\kappa_{⊥}^{r}$	8.54
$\kappa_{∥}^{r}$	10.20

**Table 2:** Maximum piezoelectric potential and displacement under forces in different directions.

Applied Force Components (nN)			Piezoelectric Potential (V)	Displacement (nm)
*x*-Axis	*y*-Axis	*z*-Axis		
0	0	100	1.85	10.2
0	100	0	0.35	48.3
100	0	0	0.29	47.3
100	100	0	0.48	67.8
100	0	100	1.85	37.6
0	100	100	1.60	49.4
100	100	100	1.60	61.6
